# Supplementary material for: End Sequence Analysis Toolkit (ESAT) expands the extractable information from single-cell RNA-seq data
Source: Genome Res. 2016 Oct;26(10):1397–410. doi: 10.1101/gr.207902.116 (PMC5052061; doi:10.1101/gr.207902.116)
Supplement: Supplemental Material [file supp_gr.207902.116_Supp_Materials.tar.gz › ESAT-esat_v0.1/doc/html/classbroad_1_1pda_1_1seq_1_1rap_1_1_genome_command_line_program.html]

ESAT: broad.pda.seq.rap.GenomeCommandLineProgram Class Reference


ESAT


- Main Page
- Related Pages
- Packages
- Classes
- Files

- Class List
- Class Index
- Class Hierarchy
- Class Members

All Classes Namespaces Files Functions Variables Enumerator Pages

- broad
- pda
- seq
- rap
- GenomeCommandLineProgram

Public Member Functions |
Public Attributes |
Protected Member Functions |
Protected Attributes |
List of all members

broad.pda.seq.rap.GenomeCommandLineProgram Class Referenceabstract

Inheritance diagram for broad.pda.seq.rap.GenomeCommandLineProgram:

[legend]

Collaboration diagram for broad.pda.seq.rap.GenomeCommandLineProgram:

[legend]

|  |  |
| --- | --- |
| Public Member Functions | |
| List< Annotation > | getRegions () |
|  | |
| Map< String, Annotation > | getRegionsMap () |
|  | |
| AnnotationList< Annotation > | getRegionSet () |
|  | |
| Annotation | getGenes () |
|  | |
| Boolean | isGenomicSpace () |
|  | |
| AlignmentModel | loadAlignmentModel (File bamFile) |
|  | |
| AlignmentModel | loadAlignmentModel (File bamFile, boolean pairedEnd) |
|  | |

|  |  |
| --- | --- |
| Public Attributes | |
| String | SIZES = "/seq/lincRNA/data/mm9/sizes" |
|  | |
| String | MASK\_FILE = "/seq/mguttman/ChIPData/MaskFiles/MM9Segments/all.mask.mouse.n36.d2.bin" |
|  | |
| double | PCT\_MASKED\_ALLOWED = 50.0 |
|  | |
| String | REGION = null |
|  | |
| String | GENE = null |
|  | |
| Integer | MAX\_FRAGMENT\_LENGTH = 10000 |
|  | |
| Integer | MIN\_MAPPING\_QUALITY = 30 |
|  | |
| String | COORD\_SPACE = "genomic" |
|  | |
| String | ANNOTATION = "/seq/lincRNA/Shari/Annotations/RefSeq\_LincV3\_ChromatinWithNames\_nonrandom\_collapsed\_overlappers.bed" |
|  | |

|  |  |
| --- | --- |
| Protected Member Functions | |
| String[] | customCommandLineValidation () |
|  | |
| void | loadCoordinateSpace () throws IOException |
|  | |
| CoordinateSpace | getCoordinateSpace () |
|  | |

|  |  |
| --- | --- |
| Protected Attributes | |
| CoordinateSpace | coordinateSpace |
|  | |

## Detailed Description

Author
:   engreitz Class used to run various analyses on sequencing data alignments in genomic or transcriptome space. Automatically filters improper pairs, chimeric reads and PCR duplicates

## Member Function Documentation

|  |  |  |  |  |
| --- | --- | --- | --- | --- |
| String [] broad.pda.seq.rap.GenomeCommandLineProgram.customCommandLineValidation | ( |  | ) |  |

protected

Here is the call graph for this function:

|  |  |  |  |  |
| --- | --- | --- | --- | --- |
| CoordinateSpace broad.pda.seq.rap.GenomeCommandLineProgram.getCoordinateSpace | ( |  | ) |  |

protected

Here is the caller graph for this function:

|  |  |  |  |  |
| --- | --- | --- | --- | --- |
| Annotation broad.pda.seq.rap.GenomeCommandLineProgram.getGenes | ( |  | ) |  |

Here is the caller graph for this function:

|  |  |  |  |  |
| --- | --- | --- | --- | --- |
| List<Annotation> broad.pda.seq.rap.GenomeCommandLineProgram.getRegions | ( |  | ) |  |

Here is the call graph for this function:

Here is the caller graph for this function:

|  |  |  |  |  |
| --- | --- | --- | --- | --- |
| AnnotationList<Annotation> broad.pda.seq.rap.GenomeCommandLineProgram.getRegionSet | ( |  | ) |  |

Here is the call graph for this function:

Here is the caller graph for this function:

|  |  |  |  |  |
| --- | --- | --- | --- | --- |
| Map<String,Annotation> broad.pda.seq.rap.GenomeCommandLineProgram.getRegionsMap | ( |  | ) |  |

Here is the caller graph for this function:

|  |  |  |  |  |
| --- | --- | --- | --- | --- |
| Boolean broad.pda.seq.rap.GenomeCommandLineProgram.isGenomicSpace | ( |  | ) |  |

|  |  |  |  |  |  |
| --- | --- | --- | --- | --- | --- |
| AlignmentModel broad.pda.seq.rap.GenomeCommandLineProgram.loadAlignmentModel | ( | File | *bamFile* | ) |  |

Here is the caller graph for this function:

|  |  |  |  |
| --- | --- | --- | --- |
| AlignmentModel broad.pda.seq.rap.GenomeCommandLineProgram.loadAlignmentModel | ( | File | *bamFile*, |
|  |  | boolean | *pairedEnd* |
|  | ) |  |  |

|  |  |  |  |  |
| --- | --- | --- | --- | --- |
| void broad.pda.seq.rap.GenomeCommandLineProgram.loadCoordinateSpace | ( |  | ) | throws IOException |

protected

Here is the call graph for this function:

Here is the caller graph for this function:

## Member Data Documentation

String broad.pda.seq.rap.GenomeCommandLineProgram.ANNOTATION = "/seq/lincRNA/Shari/Annotations/RefSeq\_LincV3\_ChromatinWithNames\_nonrandom\_collapsed\_overlappers.bed"

String broad.pda.seq.rap.GenomeCommandLineProgram.COORD\_SPACE = "genomic"

CoordinateSpace broad.pda.seq.rap.GenomeCommandLineProgram.coordinateSpace

protected

String broad.pda.seq.rap.GenomeCommandLineProgram.GENE = null

String broad.pda.seq.rap.GenomeCommandLineProgram.MASK\_FILE = "/seq/mguttman/ChIPData/MaskFiles/MM9Segments/all.mask.mouse.n36.d2.bin"

Integer broad.pda.seq.rap.GenomeCommandLineProgram.MAX\_FRAGMENT\_LENGTH = 10000

Integer broad.pda.seq.rap.GenomeCommandLineProgram.MIN\_MAPPING\_QUALITY = 30

double broad.pda.seq.rap.GenomeCommandLineProgram.PCT\_MASKED\_ALLOWED = 50.0

String broad.pda.seq.rap.GenomeCommandLineProgram.REGION = null

String broad.pda.seq.rap.GenomeCommandLineProgram.SIZES = "/seq/lincRNA/data/mm9/sizes"

---

The documentation for this class was generated from the following file:

- java/broad/pda/seq/rap/GenomeCommandLineProgram.java


---

Generated on Fri Aug 15 2014 15:11:03 for ESAT by  

 1.8.7
